# Gap junctions and hemichannels: communicating cell death in neurodevelopment and disease

**DOI:** 10.1186/s12860-016-0120-x

**Published:** 2017-01-17

**Authors:** Andrei B. Belousov, Joseph D. Fontes, Moises Freitas-Andrade, Christian C. Naus

**Affiliations:** 1 Department of Molecular & Integrative Physiology, University of Kansas Medical Center, The University of Kansas, Kansas City, KS 66160 USA; 2 Department of Biochemistry and Molecular Biology, University of Kansas Medical Center, The University of Kansas, Kansas City, KS 66160 USA; 3 Department of Cellular & Physiological Sciences, Faculty of Medicine, Life Sciences Institute, The University of British Columbia, 2350 Health Sciences Mall, Vancouver, BC V6T 1Z3 Canada

## Abstract

Gap junctions are unique membrane channels that play a significant role in intercellular communication in the developing and mature central nervous system (CNS). These channels are composed of connexin proteins that oligomerize into hexamers to form connexons or hemichannels. Many different connexins are expressed in the CNS, with some specificity with regard to the cell types in which distinct connexins are found, as well as the timepoints when they are expressed in the developing and mature CNS. Both the main neuronal Cx36 and glial Cx43 play critical roles in neurodevelopment. These connexins also mediate distinct aspects of the CNS response to pathological conditions. An imbalance in the expression, translation, trafficking and turnover of connexins, as well as mutations of connexins, can impact their function in the context of cell death in neurodevelopment and disease. With the ever-increasing understanding of connexins in the brain, therapeutic strategies could be developed to target these membrane channels in various neurological disorders.

## Background

The complexity of the mammalian central nervous system (CNS) is due in large part to the various cell types from which it is composed, as well as the different forms of cellular interactions. These include neuronal interactions via neurotransmission, as well as glial interactions mediated by direct cell-cell contact in addition to paracrine signaling (gliotransmission). Furthermore a number of glial-neuronal interactions exist, and these have been implicated in both normal information processing as well as neuronal protection in the brain. One mechanism mediating such interactions involves gap junctions (GJs), clusters of intercellular membrane channels which provide for direct cytoplasmic continuity between adjacent cells [1].

### Gap junctions and connexins in the CNS

GJs allow the passive intercellular diffusion of small molecules, such as glutamate, glutathione, glucose, adenosine triphosphate (ATP), cyclic adenosine monophosphate (cAMP), inositol 1,4,5-trisphosphate (IP_3_), and ions (Ca^2+^, Na^+^, K^+^) [2]. In addition, cells are able to control the open probability of these channels, providing a mean of including the channels in the general signaling and physiology of the cells and tissues. A single GJ channel consists of two opposing hemichannels, also known as *connexons*, which are made of six proteins called *connexins* [3]. Hemichannels can also function in their own right, having distinct roles in communicating between intracellular and extracellular compartments. Connexins are encoded by a multi-gene family consisting of 20–21 members in mammals [4].

Within the mammalian brain, the various cell types express over ten different connexins, making it a very diverse organ regarding intercellular communication. With regard to the different cell types, neurons express seven different connexins (see below), astrocytes express up to three (Cx43, Cx30, Cx26), as do oligodendrocytes (Cx32, Cx29, Cx47), microglia (Cx43, Cx32, Cx36) and endothelial cells (Cx37, Cx40, Cx43) [5–7]. These channels have distinct functions within the different cell types and their expression can change dramatically during neurodevelopment and injury (see below). GJs in oligodendrocytes have been shown to be essential for proper myelination [8], as well as potassium buffering [9]. Endothelial functions are closely regulated by junctional interactions with astrocytes; specifically important are the connexins expressed in astrocytic endfeet [10, 11]. In this context, astrocytes and endothelial cells do not form gap junctions between them, but rather the connexin in astrocytic endfeet may solely function as hemichannels.

Cx43 is the most highly expressed connexin in the brain because it is involved in extensive GJ coupling (GJC) between astrocytes, the most abundant cell type in the brain. GJ communication is also critical for the proliferation and differentiation of neural stem cells [12]. Although microglia have been reported to express Cx43 and form GJs [13], others have not observed Cx43 immunoreactivity in microglia [14, 15]; while another group showed that Cx43 does not form GJs in microglia [16], rather it forms hemichannels [17].

Connexins are expressed in both neurons and astrocytes, and are regulated by numerous factors in healthy and pathological conditions. Neuronal GJs and astrocytic GJs are regulated during development and disease. However, given the nature of the tripartite synapse, neuroglial interactions must also be considered in this context of synaptic malfunction.

### Expression and regulation of neuronal connexins in neural development and adulthood

During development, neurons of the rodent CNS express a number of different connexins. These include Cx36 [18–20], Cx30.2 [21], Cx31.1 [22], Cx40 [23], Cx45 [24] and Cx50 and Cx57 in the retina [25]. This presumably reflects the diversity of neuronal cell types, expressing a range of connexins, and/or varying functions of those connexins in the developing CNS. However, knockout of specifically Cx36 results in near complete loss of neuronal GJC in the mature CNS, indicating that it is the primary neuronal connexin [26–29]. Cx36 in various regions of rodent CNS, and Cx35 (the fish orthologue of Cx36) expressed in goldfish Mauthner cells, are often present in mixed chemical and electrical synapses. Cx36 GJs have been observed in close proximity to α-amino-3-hydroxy-5-methyl-4-isoxazolepropionic acid receptors (AMPARs) [25, 30] as well as *N*–methyl–D–aspartate (NMDA) receptors (NMDARs) [30–32]. Multiple reports have demonstrated that Cx36 GJs can be tethered to the cytoskeleton via complex formation with multiple intracellular proteins. Constituents of Cx36-interacting complexes include structural proteins, regulators of channel activity and gene transcription, as well as factors involved in protein transport, assembly and localization [33–35]. Data suggest that Cx36 may bind these proteins simultaneously at some GJs within the same neuron and the binding requires a four amino acid motif (SAYV) present in the C-terminus of the Cx36 protein [34, 35]. The interaction of Cx36 with these proteins appears to be necessary for addition into electrical synapses, since the SAYV motif is required for incorporation [36].

Cx36 is phosphorylated and its activity is influenced by a number of kinases including cAMP-dependent protein kinase (PKA), cGMP-dependent protein kinase, protein kinase C (PKC) and casein kinase II [37–39]. Ca^2+^/calmodulin-dependent protein kinase II (CaMKII) interacts with and phosphorylates Cx36 in mouse inferior olive neurons and Cx35 in synapses of teleost Mauthner cells [40, 41]. The binding of Cx36 to CaMKII may not be limited to a substrate-enzyme interaction. Rather, there is some indication that the interaction is associated with changes in expression and/or stability of the kinase. It is noteworthy that neurons of Cx36 knockout mice have reduced CaMKII levels [42].

Neuronal GJC is finely regulated among individual plaques (i.e., clusters of GJ channels). A given neuron can be coupled to a variable number of neighboring neurons and display different degrees of conductance with each of its coupled partners [32]. Similarly, binding to CaMKII [40] and the phosphorylation status of Cx36 [43] is not uniform within a neuron. Thus, the nature of signaling complexes associated with individual GJs presumably facilitates the fine-tuning of individual synapses and cell-type specific activity [43]. The interactions listed above have also been reported for non-neuronal connexins (e.g., Cx43) suggesting that modulation of GJ activity by these interactions may be a general feature [44].

Spatial and temporal variation in GJC during development of the mammalian CNS has been well documented [45–49]. The expression of Cx36 and associated GJC increase during the first two postnatal weeks in most rodent CNS regions (including the cortex and hypothalamus). This initial, relatively robust expression declines during the third and fourth postnatal weeks [18, 50, 51]. This is in contrast to other regions of the CNS, such as the spinal cord, where Cx36 expression and coupling is highest during the late embryonic period followed by a decline in the first postnatal days [18, 52, 53]. The developmental decline in GJC is paired with changes in localization of Cx36 GJs to specific neuronal subtypes in the mature CNS [54, 55]. In the developing CNS in rodents, GJC is observed between disparate neurons; glutamatergic cells (including pyramidal cells) were found to couple with interneurons [20, 56] and neurons may couple with glial cells [57]. However, in the mature CNS, Cx36 GJs are found mostly between GABAergic interneurons (GABA, γ-aminobutyric acid). It should be noted that connexins other than Cx36 also form GJs in the developing CNS [23, 58].

Despite the fact that chemical synaptic transmission is absent while Cx36 expression initiates in development [59], chemical neurotransmitter receptors regulate the developmental expression of neuronal GJC. In the rat and mouse hypothalamus and cortex, Cx36 expression is up-regulated by chronic activation of group II metabotropic glutamate receptors (mGluRs) and involves cAMP/PKA-dependent pathways. Conversely, GABA_A_ receptor activation blocks the developmental increase in Cx36 expression [51] and is dependent upon developmental depolarization and Ca^2+^/PKC-dependent signals. The developmental programs of Cx36 expression are executed using transcriptional (up-regulation) and translational (down-regulation) regulatory mechanisms [51]. These multiple mechanisms contributing to the developmental expression of Cx36 and formation of GJC likely explain the interregional differences in the developmental timing of GJC. The magnitude of the effects of neurotransmitter pathways on Cx36 expression suggests a modulatory rather than a definitive role in developmental regulation of neuronal GJC, primarily during the period when both electrical and chemical synaptic pathways are being laid down.

Regulation of Cx36 and GJC in the mature CNS occurs as well. It allows rapid modification of neuronal connectivity and signaling, including modulation of channel opening probability and alterations in connexin protein homeostasis. Similar to developmental regulation, these regulatory mechanisms are influenced by neurotransmitter signaling. For example, activation of D1 and D2 dopamine receptors, serotonin (5-HT_2_) receptors, β-adrenoreceptors and elevation of nitric oxide reduces dye coupling between rat cortical neurons within minutes [60]. Similarly, activation of β-adrenoreceptors decreases electrotonic coupling between rat hippocampal interneurons [61] and nitric oxide uncouples striatal neurons [62]. Activation of group II mGluRs in developing mouse cortical neurons induced a rapid increase followed by a decrease in Cx36 protein over a 24-h time period; Cx36 mRNA levels were unchanged during this time, suggesting regulation of protein homeostasis [63]. However, whether Cx36 channel opening probability is regulated in developing neurons is not yet known.

### The activity and regulation of Cx36 and GJC in neuronal injury and cell death

Every region of the mature CNS expresses Cx36 and has GJs, though at levels below that observed during development [54]. Transient elevation of Cx36 and GJC occurs following a wide range of neuronal insults, including ischemia [64–66], spinal cord injury and traumatic brain injury (TBI) [67–69], retinal injury [70], epilepsy [71, 72] and inflammation [73]. The up-regulation of Cx36 and GJs in neurons following injury is very rapid, occurring 1–2 h post-injury with a decline in the subsequent 24–48 h [66, 68, 69, 72, 73]. This is in stark contrast to the developmental expression program outlined earlier, which occurs on the timescale of weeks; this difference in timing suggests that disparate regulatory mechanisms may be operating in development versus injury.

The regulation of GJC expression and activity during neuronal injury is a potential therapeutic target for reducing post-injury neuronal death. Rapid upregulation of neuronal GJC and Cx36 expression was observed following multiple types of neuronal injury in adult mice [66] and coincides with the period of massive glutamate release from injured cells [74, 75]. The post-injury elevation in coupling and Cx36 was prevented by blockade of group II mGluRs and involves post-transcriptional mechanisms since no change in Cx36 mRNA levels were observed [66]. In contrast to what was observed during developmental regulation, GABA_A_ receptors were found to be only indirectly involved in reducing Cx36 expression after injury, likely via inhibition of electrical activity. Given that neuronal GJC has been shown to be, predominantly, pro-death for neurons following injury (see below), manipulation of pathways that modify expression of Cx36 might be a strategy for neuroprotection.

The morbidity and mortality of stroke is a direct result of neuronal death due primarily to ischemic injury and necrosis [76–79]. Contributing to this is excessive glutamate release from ischemic cells producing NMDAR-mediated excitotoxicity and apoptosis [80–82]. Multiple types of CNS insult beyond stroke, including TBI, epilepsy and inflammation, can produce significant neuronal death, which also involves, in part, NMDAR excitotoxicity [80, 83–86]. While studies have reported a role for GJs in cell death and survival during glutamate-mediated excitotoxicity and neuronal injury, their predominant association has been with cell death.

A few specific studies have reported a “pro-survival” role of Cx36 and GJC. For example, pharmacological blockade of GJs, using non-specific agents, augmented glutamate-induced neuronal death in mouse neuronal cortical cultures [87]. Similarly, secondary neuronal loss in the mouse retina, following infrared laser photocoagulation was most prominent 24–48 h post-injury, was increased by GJ blockade (using non-selective and relatively selective blockers for Cx36) and in Cx36 knockout mice [70]. Both studies support the notion that GJC contributes to cell survival.

The preponderance of reports, however, indicates that Cx36 GJC promotes neuronal death, independent of initiating injury. As noted earlier, sustained activation of group II mGluRs increased neuronal GJC and Cx36 expression during development; this increase amplified NMDAR-mediated excitotoxicity. Consistent with this, blockade of group II mGluRs prevented increased Cx36 expression and dampened neuronal death from excitotoxicity. These findings support a model where group II mGluRs regulate the developmental program of Cx36 GJC and by doing so, contribute significantly to death decisions in developing neurons [51]. Group II mGluR activation mediates injury-associated increases in neuronal GJC. Not surprisingly, blockade of group II mGluRs reduced injury-mediated neuronal death in multiple injury models [66, 88].

Systemic administration of NMDA induced NMDAR-mediated excitotoxicity in the forebrain of adult wild-type mice, which was prevented by co-administration of the GJC blocker mefloquine [89]. Similarly, blockade of GJC by mefloquine significantly reduced ischemic neuronal death [89] and secondary neuronal death from controlled cortical impact in mice (a model of TBI) [88]. Cx36 knockout provided the same reduction in neuronal death in both models as pharmacological blockade. Mefloquine did not provide additional survival benefit in Cx36 knockouts, suggesting the drug is working primarily through inhibition of Cx36 GJs [88, 89]. Additional studies also reported a pro-death role for neuronal GJC in NMDAR-mediated excitotoxicity and injury models [90–93]. From this and other work, a model of glutamate-mediated excitotoxicity centered on neuronal GJs as the primary determinant of magnitude of the secondary neuronal death following injury has been proposed [94, 95]. Despite the difficulty of making broad generalizations about the role of GJC in cell death, particularly in non-neuronal tissues [96, 97], blockade of GJC as a strategy to limit neuronal death following stroke, TBI or other insult remains very attractive.

### Mechanisms by which neuronal Cx36 and GJC contribute to neuronal death and survival

Understanding how neuronal GJs contribute to cell death and survival is critical, if manipulation of GJC is used as an approach to reduce neuronal damage and death in a variety of neurological diseases. Although, some of the studies discussed below were conducted with the use of non-neuronal cells, they provided an important information, which potentially may be applicable to neurons too.

Based upon numerous observations of passage of chemical substances via GJ channels, it has been proposed that the contributions of GJs to cell death and survival are by propagation between the coupled cells of, respectively, “pro-death” and “pro-survival” GJ-permeable signals [98–100]. Though the identity of these signals remains obscure, signaling molecules such as IP_3_ and reactive oxygen and nitrogen species have been proposed as “pro-death” signals [100–102]. Conversely, molecules such as those involved in energy homeostasis (glucose and ATP), and free radical scavengers (ascorbic acid and reduced glutathione) may be GJ-permeable “pro-survival” signals [96, 103]. Recently, a study was conducted in cultured neurons obtained from Cx36 knockout mice, in which the neurons were transduced with lentiviral vectors expressing one of three wild-type connexins, including neuronal Cx36 and non-neuronal Cx43 and Cx31 [93]. Ischemia and NMDAR excitotoxicity were used to induce neuronal death in those cultures. The study showed that each of the three wild-type connexins induced functional (channel-permeable) GJs and supported neuronal death. The data suggested that the role of neuronal GJs in cell death is connexin type-independent and presumably relies on channel activities of GJ complexes among neurons [93].

Another model for the role of GJC in cell death postulates that connexins are not involved in cell death mechanisms via their channel activities, but through direct or indirect regulation of transcriptional programs and apoptotic pathways [96, 104]. This model is based upon the following observations (however, mostly obtained for non-neuronal connexins). In osteocytes, Cx43 protein serves as part of a trans-membrane signal transduction pathway that alters the activity of pro-apoptotic Bcl-2 protein, Bad [105]. In non-neuronal cancer cells, Cx26 and Cx43 are co-localized with Bcl-2 proteins (Bak, Bcl-xL and Bax) and participate in cell death pathways via direct interaction with these pro-apoptotic factors [106, 107]. Overexpression of Cx43 in U251 glioblastoma cells does not increase GJC, but is pro-apoptotic [108]. During ischemia in cardiomyocytes, Cx43 serves as part of a multiprotein complex in mitochondrial membranes and controls homeostasis of mitochondria [109, 110]. Interference with expression of various connexins changes the expression of subsets of apoptotic factors (multiple studies reviewed in [96]) that presumably occurs through direct transcriptional control via the “connexin response elements” in pro-apoptotic genes [111] or via direct interaction of connexins with transcriptional regulators (e.g., β-catenin) [112]. In addition, a connexin-dependent induction of apoptosis can be connexin- and cell-specific as apoptosis in umbilical vein endothelial cells is induced by overexpression of Cx37 (but not Cx40 or Cx43), however, overexpression of Cx37 in rat NRK kidney epithelial cells is not pro-apoptotic [113]. A recent study in neuronal cultures utilized a lentiviral transduction of four mutant connexins (including various Cx36 and Cx43 mutants), each of which induced dysfunctional (channel-impermeable) GJs [93]. None of those mutant connexins supported neuronal death caused by ischemia or NMDAR excitotoxicity. This supported the notion that Cx36 unlikely plays a role in neuronal death via channel-independent mechanisms, but likley plays a role via channel-dependent mechanisms. It remains to be explored whether or not other neuronal connexins (e.g., Cx45) may contribute to neuronal death through the channel-independent mode of action.

Finally, the role of connexin hemichannels in cell death and survival has been proposed. Specifically, for the nervous system, the contribution of glial hemichannels via release of various channel-permeable “pro-death” agents has been discussed [96, 103, 114, 115]. These agents presumably include glutamate, ATP, reactive oxygen and nitrogen species. The existence and role of neuronal hemichannels in neuronal death also has been suggested based on experiments with the use of various neuronal injury models [11, 116]. However, other studies did not support the role of neuronal hemichannels in neuronal death following ischemia and NMDAR-mediated excitotoxicity [91, 93]. Moreover, the role of Cx36 hemichannels in neuroprotection via release of ATP has been suggested [117], adding an additional layer of complexity on the contribution of hemichannels in particular, and connexins in general, to neuronal death and survival.

### Expression and regulation of astroglial connexins

In the CNS, astrocytes are highly coupled to each other by GJs and play a significant role in the metabolic and trophic support of neurons [118]. These GJs are composed primarily of the channel protein Cx43, and to a lesser extent Cx30 [119] and Cx26 [120]. GJs form a functional syncytium of coupled astrocytes, contributing to spatial buffering, in dealing with elevated concentrations of extracellular potassium ions (K^+^) during increased neuronal activity; GJs assist in dispersal of K^+^ accumulated by astrocytes [121].

A major advance in understanding astrocytic GJs and connexins was made through transgenic and knockout mice (reviewed in [122]). The role of astrocytic GJs has been demonstrated in mouse hippocampal slices, with Cx43/Cx30 double knockout mice showing impaired extracellular K^+^ buffering compared to wild-type mouse slices [123]. It has also been shown that Cx43 plays a role in transient intracellular K^+^ buffering by mitochondria [124]. Pannasch et al. [10] reported key changes in astrocytic and neuronal properties in the absence of Cx43 and Cx30, revealing a major role for astrocytic networks in glutamate clearance, K^+^ buffering, and volume regulation of the extracellular space during synaptic activity. Failure to efficiently clear K^+^ and glutamate results in prolonged neuronal AMPA and NMDA currents, as well as astroglial membrane depolarization. Further clarification of the role of Cx30 was obtained by examining the hippocampus of single Cx30 knockout mice, demonstrating that Cx30 modulates astrocyte glutamate transport, thereby controlling hippocampal excitatory synaptic transmission [125]. In this case it was shown that Cx30 controls astrocytic processes at the synaptic cleft by modulating their morphology. Glutamate clearance by astrocytes was altered due to these morphological changes.

In addition to forming GJs, Cx43 also forms hemichannels, with single connexons communicating directly with the extracellular space [126]. Hemichannels predominantly exist in a closed state under normal physiological conditions, due to ambient levels of Ca^2+^ [127]. However, various cell stresses, such as hypoxia/reoxygenation and metabolic stress, have been reported to cause opening of hemichannels in cultured astrocytes [128]. Hemichannels enhance neuronal injury under ischemic and proinflammatory conditions [129, 130].

### Regulation of Cx43 in neural development

Due to the high level of GJC observed during neurodevelopment, particularly in the cortex [131, 132], it is not surprising that connexins have been shown to be involved. While neurons predominantly express Cx36 postnatally in the rat and mouse (see above), at prenatal stages neural progenitor cells (NPCs), including radial glia, are highly coupled and express Cx43 and Cx26 [47, 133–136]. Different approaches to determine the role of these connexins have been reported, including knockout of Cx43 [133, 137, 138] and knockdown of Cx43 and Cx26 [134]. While there are some variations in the phenotypes obtained, attributed in part to strain differences [138], the role of Cx43 appears to be due to adhesive functions [134], and the C-terminal region is critical for NPC migration [133, 136]. Cx26 knockdown was also shown to impede NPC migration in the developing rat cortex [134]. Cx26 has been demonstrated to be a substrate for focal adhesion kinase (FAK), or to function in stabilizing cell contacts, possibly through interactions with ZO-1 [139]. The authors suggest that FAK could act as scaffold protein, a function also suggested for Cx43. Since Cx30 is not expressed until 15 days after birth in the mouse [119] it is not considered in this context.

### The activity and regulation of Cx43 and GJC in neuronal injury and cell death

The level of GJC between astrocytes, as well as hemichannel activity [140], have been shown to be regulated by a number of factors, including neurotransmitters and neuromodulators [141–145], extracellular ion concentrations [146] and various pharmacological agents [147–150]. Astrocytic GJC and Cx43 expression are altered in various brain pathologies, including ischemia [151], stroke [152, 153], brain tumours [154], multiple sclerosis [155], brain abscess [156], Alzheimer’s disease [14, 157], and epilepsy [158, 159].

In addition, microglial response to brain injury and disease leads to the release of proinflammatory cytokines, including IL-1β and TNF-α, which impair astrocytic GJC, but enhance hemichannel activity [160]; this leads to increased neuronal injury. Short application of NMDA induces delayed neuronal injury due to excessive release of glutamate after removal of NMDA [161]. However, neurons in contact with astrocytes are protected against such glutamate toxicity [162, 163]. This neuronal protection was attributed to glutamate uptake by astrocytes [164], and as GJs are permeable to glutamate [165], GJC in astrocytes could improve glutamate uptake contributing to its dissipation, and thus to neuronal protection. In addition, GJC enables the intercellular trafficking of glucose and its metabolites through the astroglial network from blood vessels to distal neurons in an activity-dependent manner [166]. This pathway could sustain neuronal survival in pathological situations that alter energy production, such as hypoglycemia or anoxia/ischemia.

### Consideration of pannexins

A three-member family of cell membrane channels, the *pannexin(s)* (Panx 1, 2 and 3), should also be considered in the context of GJ channels and hemichannels in the CNS. Panxs were discovered due to their homology to the invertebrate GJ proteins, *innexins* [167, 168]. Panx1 and Panx2 are present in the CNS [169] and have been linked to different CNS injury models [170–172]. Because of the ubiquitous expression of Panx1 [168], it has been the most widely investigated member of the Panx family [172, 173], however, a recent study reported that Panx2 expression is not only limited to the CNS [174].

Like the connexins, the Panxs traverse the cell membrane, but these large pore\channels allow the passage of large signaling molecules (*e.g.,* ATP; glucose and glutamate) only between intra- and extracellular compartments of neurons and possibly astrocytes [175–177], but not between adjoining cells. The evidence for Panx channels not forming intercellular GJ channels, but rather the equivalent of a connexin hemichannel, has been recently addressed [178] and is attributed to the steric hindrance provided by the extracellularly glycosylated arginine residue, which interferes with the coupling of two opposing Panx channels [179, 180]. Similarly to connexins, the Panx C-terminal domain, particularly from Panx1, has been shown to interact with a host of intracellular factors under specific physiological and pathophysiological conditions [181–183].

Because Panx1 forms channels in the plasma membrane, it likely participates in non-synaptic forms of communication to regulate synaptic function under normal conditions, in addition to astrocytic Ca^2+^ wave propagation and regulation of vascular tone [184–186]. Unlike the protective effects of Cx43, however, Panx1 activation under pathological conditions is detrimental, contributing to ischemia-induced excitotoxicity and ATP-dependent cell death [176, 187–191]. Under ischemic conditions, as seen with oxygen-glucose deprivation in cortical and hippocampal slices, Panx1 channels are irreversibly activated (opened) to promote a progressive and uncontrollable depolarization of neurons, sustained increments in extracellular concentrations of glutamate and aspartate, and subsequent activation of downstream apoptotic and necrotic pathways [192]. Others have shown similar association between Panx1 activity and neuronal death, in different types of neurons [190, 193]. This suggests that Panx1-dependent cell death may be a common mechanism in injured neurons.

In addition to the anoxic depolarization mechanism associated with Panx1, it has also been implicated in contributing to inflammation [188, 194]. For example, cells undergoing apoptosis release chemotactic inflammatory factors to promote phagocytic removal of dead cells. ATP and UTP represent important signaling molecules throughout the inflammatory cascade, also thought of as danger signals that are released from damaged and necrotic cells, at least during the initial stages of ischemia [195], but also more importantly through the Panx and connexin hemichannels. Several studies have reported that Panx1-mediated ATP and UTP release is induced by caspase activity (caspase 3 and 7) in apoptotic cells [176, 187, 189]. Two potential caspase cleavage sites were identified in the C-terminal of Panx1 [189]. The C-terminal cleavage-site-B of Panx1 is evolutionarily conserved among Panx1 homologues, indicating that caspase-dependent cleavage of Panx1 and ATP release may be a conserved mechanism in apoptosis [189]. Caspase-mediated C-terminal cleavage of Panx1 results in irreversible channel opening, inducing higher if not uncontrollable extracellular release of ATP. Findings from several different organ systems collectively suggest that this irreversible activation of Panx1 leads to a cascade of maladaptive immunity, to include sustained cytokine release, improper resolution of inflammation, impaired immune cell chemotaxis, and ultimately cell death [196]. Panx1 activity has also been associated in triggering activation of the inflammasome complex [188], however, this association is not fully elucidated and maybe cell-type specific [176, 188]. Of relevance here, in an *in vivo* experimental model of retinal ischemic injury in male mice, genetic ablation of Panx1 suppresses interleukin production and protects retinal neurons from injury, highlighting the link between Panx1 and inflammation [193].

Interestingly, the reported predominance of caspase activation after ischemic injury in female mice [197] raises the intriguing possibility that the endogenous requirements for Panx1 to regulate neuronal responses to ischemic injury are different between the two sexes. With respect to connexins, whether Panx membrane channels affect connexin activity, under physiological or pathological conditions, is unknown and potentially a fruitful avenue for investigation.

### Therapeutic avenues

As discussed above, multiple studies have indicated that blockade of GJs and hemichannels provides neuroprotection in various models of neuronal injury. This suggests a possibility for using the GJ/hemichannel blockade as a novel therapeutic approach. Conceptually, blocking the propagation between the neurons of GJ-permeable toxic signals or blocking the release of toxic agents via hemichannels would create a “firebreak”, reducing the extent of cell death. This would prevent excessive neuronal death following ischemic stroke, TBI and epilepsy, significantly reducing morbidity and mortality. Because clinical trials for NMDAR antagonists as neuroprotective agents largely failed [198], development of new neuroprotective agents based on manipulation of neuronal and astroglial GJs and hemichannels should prove to be a valuable alternative approach.

## Conclusion

However, as also discussed in the present review, a number of reports suggest that blockade of GJs and hemichannels increases cell death. Clearly, the data on whether GJs are pro-death or pro-survival are conflicting and a convincing, evidence-supported explanation of this phenomenon is absent. This provides the significant barrier for translating the above-described findings to clinical practice. Without resolution of conflicting studies, manipulation of GJC in the clinic, as a novel approach to reduce neuronal death, cannot be advocated. This represents loss of a potentially extraordinary benefit to people suffering a range of brain insults. Identifying the underlying mechanisms and determining conditions for the clinical use of GJ blockers that will not compromise their strong neuroprotective effects should be the major focus of future studies.

## Abbreviations

5-HT_2_: Subfamily of serotonin receptors
AMPAR: α-amino-3-hydroxy-5-methyl-4-isoxazolepropionic acid receptor
ATP: Adenosine triphosphate
CaMKII: Ca^2+^/calmodulin-dependent protein kinase II
cAMP: Cyclic adenosine monophosphate
CNS: central nervous system
FAK: Focal adhesion kinase
GABA: γ-aminobutyric acid
GJ: Gap junction
GJC: Gap junction coupling
IP_3_: Inositol 1,4,5-trisphosphate
mGluR: Metabotropic glutamate receptor
NMDAR: *N*–methyl–D–aspartate receptor
NPC: Neural progenitor cell
Panx: Pannexin
PKA: Protein kinase A
PKC: Protein kinase C
UTP: Uridine triphosphate

## References

[1] Simon AM, Goodenough DA (1998). Diverse functions of vertebrate gap junctions. Trends Cell Biol.

[2] Alexander DB, Goldberg GS (2003). Transfer of biologically important molecules between cells through gap junction channels. Curr Med Chem.

[3] Sohl G, Willecke K (2004). Gap junctions and the connexin protein family. Cardiovasc Res.

[4] Willecke K, Eiberger J, Degen J, Eckardt D, Romualdi A, Guldenagel M, Deutsch U, Sohl G (2002). Structural and functional diversity of connexin genes in the mouse and human genome. Biol Chem.

[5] De Bock M, Wang N, Decrock E, Bol M, Gadicherla AK, Culot M, Cecchelli R, Bultynck G, Leybaert L (2013). Endothelial calcium dynamics, connexin channels and blood–brain barrier function. Prog Neurobiol.

[6] De Bock M, Culot M, Wang N, Bol M, Decrock E, De Vuyst E, da Costa A, Dauwe I, Vinken M, Simon AM (2011). Connexin channels provide a target to manipulate brain endothelial calcium dynamics and blood–brain barrier permeability. J Cereb Blood Flow Metab.

[7] Ransom B, Giaume C. Gap junctions and hemichannels. In: Kettenmann H, Ransom B, editors. Neuroglia. New York: Oxford University Press; 2012.

[8] Menichella DM, Goodenough DA, Sirkowski E, Scherer SS, Paul DL (2003). Connexins are critical for normal myelination in the CNS. J Neurosci.

[9] Menichella DM, Majdan M, Awatramani R, Goodenough DA, Sirkowski E, Scherer SS, Paul DL (2006). Genetic and physiological evidence that oligodendrocyte gap junctions contribute to spatial buffering of potassium released during neuronal activity. J Neurosci.

[10] Pannasch U, Vargova L, Reingruber J, Ezan P, Holcman D, Giaume C, Sykova E, Rouach N (2011). Astroglial networks scale synaptic activity and plasticity. Proc Natl Acad Sci U S A.

[11] Froger N, Orellana JA, Calvo CF, Amigou E, Kozoriz MG, Naus CC, Saez JC, Giaume C (2010). Inhibition of cytokine-induced connexin43 hemichannel activity in astrocytes is neuroprotective. Mol Cell Neurosci.

[12] Worsdorfer P, Maxeiner S, Markopoulos C, Kirfel G, Wulf V, Auth T, Urschel S, von Maltzahn J, Willecke K (2008). Connexin expression and functional analysis of gap junctional communication in mouse embryonic stem cells. Stem Cells.

[13] Eugenin EA, Eckardt D, Theis M, Willecke K, Bennett MV, Saez JC (2001). Microglia at brain stab wounds express connexin 43 and in vitro form functional gap junctions after treatment with interferon-gamma and tumor necrosis factor-alpha. Proc Natl Acad Sci U S A.

[14] Mei X, Ezan P, Giaume C, Koulakoff A (2010). Astroglial connexin immunoreactivity is specifically altered at beta-amyloid plaques in beta-amyloid precursor protein/presenilin1 mice. Neuroscience.

[15] Theodoric N, Bechberger JF, Naus CC, Sin WC (2012). Role of gap junction protein connexin43 in astrogliosis induced by brain injury. PLoS One.

[16] Wasseff SK, Scherer SS (2014). Activated microglia do not form functional gap junctions in vivo. J Neuroimmunol.

[17] Takeuchi H, Jin S, Wang J, Zhang G, Kawanokuchi J, Kuno R, Sonobe Y, Mizuno T, Suzumura A (2006). Tumor necrosis factor-alpha induces neurotoxicity via glutamate release from hemichannels of activated microglia in an autocrine manner. J Biol Chem.

[18] Belluardo N, Mudo G, Trovato-Salinaro A, Le Gurun S, Charollais A, Serre-Beinier V, Amato G, Haefliger JA, Meda P, Condorelli DF (2000). Expression of connexin36 in the adult and developing rat brain. Brain Res.

[19] Rash JE, Kamasawa N, Davidson KG, Yasumura T, Pereda AE, Nagy JI (2012). Connexin composition in apposed gap junction hemiplaques revealed by matched double-replica freeze-fracture replica immunogold labeling. J Membr Biol.

[20] Venance L, Rozov A, Blatow M, Burnashev N, Feldmeyer D, Monyer H (2000). Connexin expression in electrically coupled postnatal rat brain neurons. Proc Natl Acad Sci U S A.

[21] Kreuzberg MM, Deuchars J, Weiss E, Schober A, Sonntag S, Wellershaus K, Draguhn A, Willecke K (2008). Expression of connexin30.2 in interneurons of the central nervous system in the mouse. Mol Cell Neurosci.

[22] Dere E, Zheng-Fischhofer Q, Viggiano D, Gironi Carnevale UA, Ruocco LA, Zlomuzica A, Schnichels M, Willecke K, Huston JP, Sadile AG (2008). Connexin31.1 deficiency in the mouse impairs object memory and modulates open-field exploration, acetylcholine esterase levels in the striatum, and cAMP response element-binding protein levels in the striatum and piriform cortex. Neuroscience.

[23] Personius KE, Chang Q, Mentis GZ, O’Donovan MJ, Balice-Gordon RJ (2007). Reduced gap junctional coupling leads to uncorrelated motor neuron firing and precocious neuromuscular synapse elimination. Proc Natl Acad Sci U S A.

[24] Li X, Kamasawa N, Ciolofan C, Olson CO, Lu S, Davidson KG, Yasumura T, Shigemoto R, Rash JE, Nagy JI (2008). Connexin45-containing neuronal gap junctions in rodent retina also contain connexin36 in both apposing hemiplaques, forming bihomotypic gap junctions, with scaffolding contributed by zonula occludens-1. J Neurosci.

[25] Puller C, de Sevilla Muller LP, Janssen-Bienhold U, Haverkamp S (2009). ZO-1 and the spatial organization of gap junctions and glutamate receptors in the outer plexiform layer of the mammalian retina. J Neurosci.

[26] De Zeeuw CI, Chorev E, Devor A, Manor Y, Van Der Giessen RS, De Jeu MT, Hoogenraad CC, Bijman J, Ruigrok TJ, French P (2003). Deformation of network connectivity in the inferior olive of connexin 36-deficient mice is compensated by morphological and electrophysiological changes at the single neuron level. J Neurosci.

[27] Deans MR, Gibson JR, Sellitto C, Connors BW, Paul DL (2001). Synchronous activity of inhibitory networks in neocortex requires electrical synapses containing connexin36. Neuron.

[28] Hormuzdi SG, Pais I, LeBeau FE, Towers SK, Rozov A, Buhl EH, Whittington MA, Monyer H (2001). Impaired electrical signaling disrupts gamma frequency oscillations in connexin 36-deficient mice. Neuron.

[29] Long MA, Deans MR, Paul DL, Connors BW (2002). Rhythmicity without synchrony in the electrically uncoupled inferior olive. J Neurosci.

[30] Hamzei-Sichani F, Davidson KG, Yasumura T, Janssen WG, Wearne SL, Hof PR, Traub RD, Gutierrez R, Ottersen OP, Rash JE (2012). Mixed electrical-chemical synapses in adult rat hippocampus are primarily glutamatergic and coupled by connexin-36. Front Neuroanat.

[31] Nagy JI (2012). Evidence for connexin36 localization at hippocampal mossy fiber terminals suggesting mixed chemical/electrical transmission by granule cells. Brain Res.

[32] Hoge GJ, Davidson KG, Yasumura T, Castillo PE, Rash JE, Pereda AE (2011). The extent and strength of electrical coupling between inferior olivary neurons is heterogeneous. J Neurophysiol.

[33] Ciolofan C, Li XB, Olson C, Kamasawa N, Gebhardt BR, Yasumura T, Morita M, Rash JE, Nagy JI (2006). Association of connexin36 and zonula occludens-1 with zonula occludens-2 and the transcription factor zonula occludens-1-associated nucleic acid-binding protein at neuronal gap junctions in rodent retina. Neuroscience.

[34] Li X, Lu S, Nagy JI (2009). Direct association of connexin36 with zonula occludens-2 and zonula occludens-3. Neurochem Int.

[35] Li X, Lynn BD, Nagy JI (2012). The effector and scaffolding proteins AF6 and MUPP1 interact with connexin36 and localize at gap junctions that form electrical synapses in rodent brain. Eur J Neurosci.

[36] Helbig I, Sammler E, Eliava M, Bolshakov AP, Rozov A, Bruzzone R, Monyer H, Hormuzdi SG (2010). In vivo evidence for the involvement of the carboxy terminal domain in assembling connexin 36 at the electrical synapse. Mol Cell Neurosci.

[37] Kothmann WW, Li X, Burr GS, O’Brien J (2007). Connexin 35/36 is phosphorylated at regulatory sites in the retina. Vis Neurosci.

[38] Patel LS, Mitchell CK, Dubinsky WP, O’Brien J (2006). Regulation of gap junction coupling through the neuronal connexin Cx35 by nitric oxide and cGMP. Cell Commun Adhes.

[39] Urschel S, Hoher T, Schubert T, Alev C, Sohl G, Worsdorfer P, Asahara T, Dermietzel R, Weiler R, Willecke K (2006). Protein kinase A-mediated phosphorylation of connexin36 in mouse retina results in decreased gap junctional communication between AII amacrine cells. J Biol Chem.

[40] Flores CE, Cachope R, Nannapaneni S, Ene S, Nairn AC, Pereda AE (2010). Variability of distribution of Ca(2+)/calmodulin-dependent kinase II at mixed synapses on the mauthner cell: colocalization and association with connexin 35. J Neurosci.

[41] Alev C, Urschel S, Sonntag S, Zoidl G, Fort AG, Hoher T, Matsubara M, Willecke K, Spray DC, Dermietzel R (2008). The neuronal connexin36 interacts with and is phosphorylated by CaMKII in a way similar to CaMKII interaction with glutamate receptors. Proc Natl Acad Sci U S A.

[42] Zlomuzica A, Viggiano D, Degen J, Binder S, Ruocco LA, Sadile AG, Willecke K, Huston JP, Dere E (2012). Behavioral alterations and changes in Ca/calmodulin kinase II levels in the striatum of connexin36 deficient mice. Behav Brain Res.

[43] Kothmann WW, Massey SC, O’Brien J (2009). Dopamine-stimulated dephosphorylation of connexin 36 mediates AII amacrine cell uncoupling. J Neurosci.

[44] Herve JC, Derangeon M, Sarrouilhe D, Giepmans BN, Bourmeyster N (2012). Gap junctional channels are parts of multiprotein complexes. Biochim Biophys Acta.

[45] Eugenin EA, Basilio D, Saez JC, Orellana JA, Raine CS, Bukauskas F, Bennett MV, Berman JW (2012). The role of gap junction channels during physiologic and pathologic conditions of the human central nervous system. J Neuroimmune Pharmacol.

[46] Dermietzel R, Traub O, Hwang TK, Beyer E, Bennett MV, Spray DC, Willecke K (1989). Differential expression of three gap junction proteins in developing and mature brain tissues. Proc Natl Acad Sci U S A.

[47] Cina C, Bechberger JF, Ozog MA, Naus CC (2007). Expression of connexins in embryonic mouse neocortical development. J Comp Neurol.

[48] Belliveau DJ, Kidder GM, Naus CCG (1991). Expression of gap junction genes during postnatal neural development. Dev Genet.

[49] Belliveau DJ, Naus CCG (1995). Differential localization of gap junction mRNAs in developing rat brain. Dev Neurosci.

[50] Arumugam H, Liu X, Colombo PJ, Corriveau RA, Belousov AB (2005). NMDA receptors regulate developmental gap junction uncoupling via CREB signaling. Nat Neurosci.

[51] Park W-M, Wang Y, Park S, Denisova JV, Fontes JD, Belousov AB (2011). Interplay of chemical neurotransmitters regulates developmental increase in electrical synapses. J Neurosci.

[52] Mentis GZ, Diaz E, Moran LB, Navarrete R (2002). Increased incidence of gap junctional coupling between spinal motoneurones following transient blockade of NMDA receptors in neonatal rats. J Physiol.

[53] Pastor AM, Mentis GZ, De La Cruz RR, Diaz E, Navarrete R (2003). Increased electrotonic coupling in spinal motoneurons after transient botulinum neurotoxin paralysis in the neonatal rat. J Neurophysiol.

[54] Connors BW. Chapter 6: Electrical signaling with neuronal gap junctions**.** In: Harris A, Locke D, editors. Connexins: A Guide*.* New York: Humana Press; 2009. pp. 143–64.

[55] Hestrin S, Galarreta M (2005). Electrical synapses define networks of neocortical GABAergic neurons. Trends Neurosci.

[56] Meyer AH, Katona I, Blatow M, Rozov A, Monyer H (2002). In vivo labeling of parvalbumin-positive interneurons and analysis of electrical coupling in identified neurons. J Neurosci.

[57] Alvarez-Maubecin V, Garcia-Hernandez F, Williams JT, Van Bockstaele EJ (2000). Functional coupling between neurons and glia. J Neurosci.

[58] Yu YC, He S, Chen S, Fu Y, Brown KN, Yao XH, Ma J, Gao KP, Sosinsky GE, Huang K (2012). Preferential electrical coupling regulates neocortical lineage-dependent microcircuit assembly. Nature.

[59] Ben-Ari Y (2001). Developing networks play a similar melody. Trends Neurosci.

[60] Roerig B, Feller MB (2000). Neurotransmitters and gap junctions in developing neural circuits. Brain Res Brain Res Rev.

[61] Zsiros V, Maccaferri G (2008). Noradrenergic modulation of electrical coupling in GABAergic networks of the hippocampus. J Neurosci.

[62] O’Donnell P, Grace AA (1997). Cortical afferents modulate striatal gap junction permeability via nitric oxide. Neuroscience.

[63] Song J-H, Wang Y, Fontes JD, Belousov AB (2012). Regulation of connexin 36 expression during development. Neurosci Lett.

[64] de Pina-Benabou MH, Szostak V, Kyrozis A, Rempe D, Uziel D, Urban-Maldonado M, Benabou S, Spray DC, Federoff HJ, Stanton PK (2005). Blockade of gap junctions in vivo provides neuroprotection after perinatal global ischemia. Stroke.

[65] Oguro K, Jover T, Tanaka H, Lin Y, Kojima T, Oguro N, Grooms SY, Bennett MV, Zukin RS (2001). Global ischemia-induced increases in the gap junctional proteins connexin 32 (Cx32) and Cx36 in hippocampus and enhanced vulnerability of Cx32 knock-out mice. J Neurosci.

[66] Wang Y, Song J-H, Denisova JV, Park W-M, Fontes JD, Belousov AB (2012). Neuronal gap junction coupling is regulated by glutamate and plays critical role in cell death during neuronal injury. J Neurosci.

[67] Chang Q, Pereda A, Pinter MJ, Balice-Gordon RJ (2000). Nerve injury induces gap junctional coupling among axotomized adult motor neurons. J Neurosci.

[68] Frantseva MV, Kokarovtseva L, Naus CG, Carlen PL, MacFabe D, Perez Velazquez JL (2002). Specific gap junctions enhance the neuronal vulnerability to brain traumatic injury. J Neurosci.

[69] Ohsumi A, Nawashiro H, Otani N, Ooigawa H, Toyooka T, Yano A, Nomura N, Shima K (2006). Alteration of gap junction proteins (connexins) following lateral fluid percussion injury in rats. Acta Neurochir Suppl.

[70] Striedinger K, Petrasch-Parwez E, Zoidl G, Napirei M, Meier C, Eysel UT, Dermietzel R (2005). Loss of connexin36 increases retinal cell vulnerability to secondary cell loss. Eur J Neurosci.

[71] Gajda Z, Gyengesi E, Hermesz E, Ali KS, Szente M (2003). Involvement of gap junctions in the manifestation and control of the duration of seizures in rats in vivo. Epilepsia.

[72] Perez Velazquez JL, Valiante TA, Carlen PL (1994). Modulation of gap junctional mechanisms during calcium-free induced field burst activity: a possible role for electrotonic coupling in epileptogenesis. J Neurosci.

[73] Garrett FG, Durham PL (2008). Differential expression of connexins in trigeminal ganglion neurons and satellite glial cells in response to chronic or acute joint inflammation. Neuron Glia Biol.

[74] Guyot LL, Diaz FG, O’Regan MH, McLeod S, Park H, Phillis JW (2001). Real-time measurement of glutamate release from the ischemic penumbra of the rat cerebral cortex using a focal middle cerebral artery occlusion model. Neurosci Lett.

[75] Stoffel M, Plesnila N, Eriskat J, Furst M, Baethmann A (2002). Release of excitatory amino acids in the penumbra of a focal cortical necrosis. J Neurotrauma.

[76] Fortuna S, Pestalozza S, Lorenzini P, Bisso GM, Morelli L, Michalek H (1997). Transient global brain hypoxia-ischemia in adult rats: neuronal damage, glial proliferation, and alterations in inositol phospholipid hydrolysis. Neurochem Int.

[77] Kirino T, Tamura A, Sano K (1984). Delayed neuronal death in the rat hippocampus following transient forebrain ischemia. Acta Neuropathol.

[78] Petito CK, Feldmann E, Pulsinelli WA, Plum F (1987). Delayed hippocampal damage in humans following cardiorespiratory arrest. Neurology.

[79] Xu BN, Yabuki A, Mishina H, Miyazaki M, Maeda M, Ishii S (1993). Pathophysiology of brain swelling after acute experimental brain compression and decompression. Neurosurgery.

[80] Arundine M, Tymianski M (2004). Molecular mechanisms of glutamate-dependent neurodegeneration in ischemia and traumatic brain injury. Cell Mol Life Sci.

[81] Reyes M, Reyes A, Opitz T, Kapin MA, Stanton PK (1998). Eliprodil, a non-competitive, NR2B-selective NMDA antagonist, protects pyramidal neurons in hippocampal slices from hypoxic/ischemic damage. Brain Res.

[82] Lees KR (1997). Cerestat and other NMDA antagonists in ischemic stroke. Neurology.

[83] Biegon A, Fry PA, Paden CM, Alexandrovich A, Tsenter J, Shohami E (2004). Dynamic changes in N-methyl-D-aspartate receptors after closed head injury in mice: Implications for treatment of neurological and cognitive deficits. Proc Natl Acad Sci U S A.

[84] Giza CC, Maria NS, Hovda DA (2006). N-methyl-D-aspartate receptor subunit changes after traumatic injury to the developing brain. J Neurotrauma.

[85] Faden AI, Demediuk P, Panter SS, Vink R (1989). The role of excitatory amino acids and NMDA receptors in traumatic brain injury. Science.

[86] Rogawski MA (1992). The NMDA receptor, NMDA antagonists and epilepsy therapy. A status report. Drugs.

[87] Ozog MA, Siushansian R, Naus CC (2002). Blocked gap junctional coupling increases glutamate-induced neurotoxicity in neuron-astrocyte co-cultures. J Neuropathol Exp Neurol.

[88] Belousov AB, Wang Y, Song J-H, Denisova JV, Berman NE, Fontes JD (2012). Neuronal gap junctions play a role in the secondary neuronal death following controlled cortical impact. Neurosci Lett.

[89] Wang Y, Denisova JV, Kang KS, Fontes JD, Zhu BT, Belousov AB (2010). Neuronal gap junctions are required for NMDA receptor-mediated excitotoxicity: implications in ischemic stroke. J Neurophysiol.

[90] Cusato K, Bosco A, Rozental R, Guimaraes CA, Reese BE, Linden R, Spray DC (2003). Gap junctions mediate bystander cell death in developing retina. J Neurosci.

[91] de Rivero Vaccari JC, Corriveau RA, Belousov AB (2007). Gap junctions are required for NMDA receptor-dependent cell death in developing neurons. J Neurophysiol.

[92] Frantseva MV, Kokarovtseva L, Perez Velazquez JL (2002). Ischemia-induced brain damage depends on specific gap-junctional coupling. J Cereb Blood Flow Metab.

[93] Fontes JD, Ramsey J, Polk JM, Koop A, Denisova JV, Belousov AB (2015). Death of neurons following injury requires conductive neuronal gap junction channels but not a specific connexin. PLoS One.

[94] Belousov AB, Fontes JD (2013). Neuronal gap junctions: making and breaking connections during development and injury. Trends Neurosci.

[95] Belousov AB, Fontes JD (2014). Neuronal gap junction coupling as the primary determinant of the extent of glutamate-mediated excitotoxicity. J Neural Transm.

[96] Decrock E, Vinken M, De Vuyst E, Krysko DV, D’Herde K, Vanhaecke T, Vandenabeele P, Rogiers V, Leybaert L (2009). Connexin-related signaling in cell death: to live or let die?. Cell Death Differ.

[97] Perez Velazquez JL, Frantseva MV, Naus CC (2003). Gap junctions and neuronal injury: protectants or executioners?. Neuroscientist.

[98] Udawatte C, Ripps H (2005). The spread of apoptosis through gap-junctional channels in BHK cells transfected with Cx32. Apoptosis.

[99] Cusato K, Ripps H, Zakevicius J, Spray DC (2006). Gap junctions remain open during cytochrome c-induced cell death: relationship of conductance to ‘bystander’ cell killing. Cell Death Differ.

[100] Decrock E, De Bock M, Wang N, Gadicherla AK, Bol M, Delvaeye T, Vandenabeele P, Vinken M, Bultynck G, Krysko DV (2013). IP3, a small molecule with a powerful message. Biochim Biophys Acta.

[101] Decrock E, Krysko DV, Vinken M, Kaczmarek A, Crispino G, Bol M, Wang N, De Bock M, De Vuyst E, Naus CC (2012). Transfer of IP3 through gap junctions is critical, but not sufficient, for the spread of apoptosis. Cell Death Differ.

[102] Monaco G, Decrock E, Akl H, Ponsaerts R, Vervliet T, Luyten T, De Maeyer M, Missiaen L, Distelhorst CW, De Smedt H (2012). Selective regulation of IP3-receptor-mediated Ca2+ signaling and apoptosis by the BH4 domain of Bcl-2 versus Bcl-Xl. Cell Death Differ.

[103] Decrock E, Vinken M, Bol M, D’Herde K, Rogiers V, Vandenabeele P, Krysko DV, Bultynck G, Leybaert L (2011). Calcium and connexin-based intercellular communication, a deadly catch?. Cell Calcium.

[104] Vinken M, Decrock E, Leybaert L, Bultynck G, Himpens B, Vanhaecke T, Rogiers V (2012). Non-channel functions of connexins in cell growth and cell death. Biochim Biophys Acta.

[105] Plotkin LI, Manolagas SC, Bellido T (2002). Transduction of cell survival signals by connexin-43 hemichannels. J Biol Chem.

[106] Kanczuga-Koda L, Sulkowski S, Koda M, Skrzydlewska E, Sulkowska M (2005). Connexin 26 correlates with Bcl-xL and Bax proteins expression in colorectal cancer. World J Gastroenterol.

[107] Kanczuga-Koda L, Sulkowski S, Tomaszewski J, Koda M, Sulkowska M, Przystupa W, Golaszewska J, Baltaziak M (2005). Connexins 26 and 43 correlate with Bak, but not with Bcl-2 protein in breast cancer. Oncol Rep.

[108] Huang RP, Hossain MZ, Huang R, Gano J, Fan Y, Boynton AL (2001). Connexin 43 (cx43) enhances chemotherapy-induced apoptosis in human glioblastoma cells. Int J Cancer.

[109] Ruiz-Meana M, Rodriguez-Sinovas A, Cabestrero A, Boengler K, Heusch G, Garcia-Dorado D (2008). Mitochondrial connexin43 as a new player in the pathophysiology of myocardial ischaemia-reperfusion injury. Cardiovasc Res.

[110] Goubaeva F, Mikami M, Giardina S, Ding B, Abe J, Yang J (2007). Cardiac mitochondrial connexin 43 regulates apoptosis. Biochem Biophys Res Commun.

[111] Stains JP, Lecanda F, Screen J, Towler DA, Civitelli R (2003). Gap junctional communication modulates gene transcription by altering the recruitment of Sp1 and Sp3 to connexin-response elements in osteoblast promoters. J Biol Chem.

[112] Ai Z, Fischer A, Spray DC, Brown AM, Fishman GI (2000). Wnt-1 regulation of connexin43 in cardiac myocytes. J Clin Invest.

[113] Seul KH, Kang KY, Lee KS, Kim SH, Beyer EC (2004). Adenoviral delivery of human connexin37 induces endothelial cell death through apoptosis. Biochem Biophys Res Commun.

[114] Saez JC, Schalper KA, Retamal MA, Orellana JA, Shoji KF, Bennett MV (2010). Cell membrane permeabilization via connexin hemichannels in living and dying cells. Exp Cell Res.

[115] Davidson JO, Green CR, LF BN, O’Carroll SJ, Fraser M, Bennet L, Jan Gunn A (2012). Connexin hemichannel blockade improves outcomes in a model of fetal ischemia. Ann Neurol.

[116] Orellana JA, Shoji KF, Abudara V, Ezan P, Amigou E, Saez PJ, Jiang JX, Naus CC, Saez JC, Giaume C (2011). Amyloid beta-induced death in neurons involves glial and neuronal hemichannels. J Neurosci.

[117] Schock SC, Leblanc D, Hakim AM, Thompson CS (2008). ATP release by way of connexin 36 hemichannels mediates ischemic tolerance in vitro. Biochem Biophys Res Commun.

[118] Jensen AM, Chiu S, Murphy S (1993). Astrocyte Networks. Astrocytes: Pharmacology and Function.

[119] Nagy JI, Patel D, Ochalski PA, Stelmack GL (1999). Connexin30 in rodent, cat and human brain: selective expression in gray matter astrocytes, co-localization with connexin43 at gap junctions and late developmental appearance. Neuroscience.

[120] Nagy JI, Li X, Rempel J, Stelmack G, Patel D, Staines WA, Yasumura T, Rash JE (2001). Connexin26 in adult rodent central nervous system: demonstration at astrocytic gap junctions and colocalization with connexin30 and connexin43. J Comp Neurol.

[121] Walz W, Hertz L (1983). Functional interactions between neurons and astrocytes. II. Potassium homeostasis at the cellular level. Prog Neurobiol.

[122] Giaume C, Theis M (2010). Pharmacological and genetic approaches to study connexin-mediated channels in glial cells of the central nervous system. Brain Res Rev.

[123] Wallraff A, Kohling R, Heinemann U, Theis M, Willecke K, Steinhauser C (2006). The impact of astrocytic gap junctional coupling on potassium buffering in the hippocampus. J Neurosci.

[124] Kozoriz MG, Church J, Ozog MA, Naus CC, Krebs C (2010). Temporary sequestration of potassium by mitochondria in astrocytes. J Biol Chem.

[125] Pannasch U, Freche D, Dallerac G, Ghezali G, Escartin C, Ezan P, Cohen-Salmon M, Benchenane K, Abudara V, Dufour A (2014). Connexin 30 sets synaptic strength by controlling astroglial synapse invasion. Nat Neurosci.

[126] Goodenough DA, Paul DL (2003). Beyond the gap: functions of unpaired connexon channels. Nat Rev Mol Cell Biol.

[127] Li HY, Liu TF, Lazrak A, Peracchia C, Goldberg GS, Lampe PD, Johnson R (1996). Properties and regulation of gap junctional hemichannels in the plasma membranes of cultured cells. J Cell Biol.

[128] Le HT, Sin WC, Lozinsky S, Bechberger J, Vega JL, Guo XQ, Saez JC, Naus CC (2014). Gap junction intercellular communication mediated by connexin43 in astrocytes is essential for their resistance to oxidative stress. J Biol Chem.

[129] Contreras JE, Sanchez HA, Veliz LP, Bukauskas FF, Bennett MV, Saez JC (2004). Role of connexin-based gap junction channels and hemichannels in ischemia-induced cell death in nervous tissue. Brain Res Brain Res Rev.

[130] Orellana JA, von Bernhardi R, Giaume C, Saez JC (2012). Glial hemichannels and their involvement in aging and neurodegenerative diseases. Rev Neurosci.

[131] Yuste R, Peinado A, Katz LC (1992). Neuronal domains in developing neocortex. Science.

[132] Peinado A, Yuste R, Katz LC (1993). Extensive dye coupling between rat neocortical neurons during the period of circuit formation. Neuron.

[133] Cina C, Maass K, Theis M, Willecke K, Bechberger JF, Naus CC (2009). Involvement of the cytoplasmic C-terminal domain of connexin43 in neuronal migration. J Neurosci.

[134] Elias LA, Wang DD, Kriegstein AR (2007). Gap junction adhesion is necessary for radial migration in the neocortex. Nature.

[135] Elias LAB, Kriegstein AR (2010). Gap junctions: Multifaceted regulators of embryonic cortical development. Trends Neurosci.

[136] Elias LAB, Turmaine M, Parnavelas JG, Kriegstein AR (2010). Connexin43 mediates the tangential to radial migratory switch in ventrally derived cortical interneurons. J Neurosci.

[137] Fushiki S, Perez Velazquez JL, Zhang L, Bechberger JF, Carlen PL, Naus CC (2003). Changes in neuronal migration in neocortex of connexin43 null mutant mice. J Neuropathol Exp Neurol.

[138] Wiencken-Barger AE, Djukic B, Casper KB, McCarthy KD (2007). A role for Connexin43 during neurodevelopment. GLIA.

[139] Valiente M, Ciceri G, Rico B, Marin O (2011). Focal adhesion kinase modulates radial glia-dependent neuronal migration through connexin-26. J Neurosci.

[140] Giaume C, Leybaert L, Naus CC, Saez JC (2013). Connexin and pannexin hemichannels in brain glial cells: properties, pharmacology, and roles. Front Pharmacol.

[141] Ochalski PAY, Sawchuk MA, Hertzberg EL, Nagy JI (1995). Astrocytic gap junction removal, connexin43 redistribution, and epitope masking at excitatory amino acid lesion sites in rat brain. GLIA.

[142] Vukelic JI, Yamamoto T, Hertzberg EL, Nagy JI (1991). Depletion of connexin43-immunoreactivity in astrocytes after kainic acid-induced lesions in rat brain. NeurosciLett.

[143] Giaume C, Cordier J, Glowinski J (1992). Endothelins inhibit junctional permeability in cultured mouse astrocytes. Euro J Neurosci.

[144] Giaume C, Briley M, Marien M (1994). Noradrenergic control of gap junction permeability in cultured striatal astrocytes. Noradrenergic Mechanisms in Parkinson’s Disease.

[145] Giaume C, Marin P, Cordier J, Glowinski J, Premont J (1991). Adrenergic regulation of intercellular communications between cultured striatal astrocytes from the mouse. Proc Natl Acad Sci U S A.

[146] Enkvist MO, McCarthy KD (1994). Astroglial gap junction communication is increased by treatment with either glutamate or high K+ concentration. J Neurochem.

[147] Konietzko U, Muller CM (1994). Astrocytic dye coupling in rat hippocampus: Topography, developmental onset, and modulation by protein kinase C. Hippocampus.

[148] MacVicar BA, Jahnsen H (1985). Uncoupling of CA3 pyramidal neurons by propionate. Brain Res.

[149] Mantz J, Cordier J, Giaume C (1993). Effects of general anesthetics on intercellular communications mediated by gap junctions between astrocytes in primary culture. Anesthesiology.

[150] Anders JJ (1988). Lactic acid inhibition of gap junctional intercellular communication in in vitro astrocytes as measured by fluorescence recovery after laser photobleaching. GLIA.

[151] Hossain MZ, Peeling J, Sutherland GR, Hertzberg EL, Nagy JI (1994). Ischemia-induced cellular redistribution of the astrocytic gap junctional protein connexin43 in rat brain. Brain Res.

[152] Nakase T, Yoshida Y, Nagata K (2006). Enhanced connexin 43 immunoreactivity in penumbral areas in the human brain following ischemia. Glia.

[153] Haupt C, Witte OW, Frahm C (2007). Temporal profile of connexin 43 expression after photothrombotic lesion in rat brain. Neuroscience.

[154] Kolar K, Freitas-Andrade M, Bechberger JF, Krishnan H, Goldberg GS, Naus CC, Sin WC (2015). Podoplanin: a marker for reactive gliosis in gliomas and brain injury. J Neuropathol Exp Neurol.

[155] Brand-Schieber E, Werner P, Iacobas DA, Iacobas S, Beelitz M, Lowery SL, Spray DC, Scemes E (2005). Connexin43, the major gap junction protein of astrocytes, is down-regulated in inflamed white matter in an animal model of multiple sclerosis. J Neurosci Res.

[156] Karpuk N, Burkovetskaya M, Fritz T, Angle A, Kielian T (2011). Neuroinflammation leads to region-dependent alterations in astrocyte gap junction communication and hemichannel activity. J Neurosci.

[157] Koulakoff A, Mei X, Orellana JA, Saez JC, Giaume C (2012). Glial connexin expression and function in the context of Alzheimer’s disease. Biochim Biophys Acta.

[158] Naus CC, Bechberger JF, Paul DL (1991). Gap junction gene expression in human seizure disorder. Exp Neurol.

[159] Elisevich K, Rempel SA, Smith BJ, Edvardsen K (1997). Hippocampal connexin 43 expression in human complex partial seizure disorder. Exp Neurol.

[160] Retamal MA, Schalper KA, Shoji KF, Orellana JA, Bennett MV, Saez JC (2007). Possible involvement of different connexin43 domains in plasma membrane permeabilization induced by ischemia-reperfusion. J Membr Biol.

[161] Hartley DM, Choi DW (1989). Delayed rescue of N-methyl-D-aspartate receptor-mediated neuronal injury in cortical culture. J Pharmacol Exp Ther.

[162] Mattson MP, Rychlik B (1990). Glia protect hippocampal neurons against excitatory amino acid-induced degeneration: involvement of fibroblast growth factor. Int J Dev Neurosci.

[163] Rosenberg PA, Aizenman E (1989). Hundred-fold increase in neuronal vulnerability to glutamate toxicity in astrocyte-poor cultures of rat cerebral cortex [published erratum appears in Neurosci Lett 1990 Aug 24;116(3): 399]. Neurosci Lett.

[164] Rosenberg PA (1991). Accumulation of extracellular glutamate and neuronal death in astrocyte-poor cortical cultures exposed to glutamine. GLIA.

[165] Goldberg GS, Lampe PD, Nicholson BJ (1999). Selective transfer of endogenous metabolites through gap junctions composed of different connexins. Nat Cell Biol.

[166] Rouach N, Koulakoff A, Abudara V, Willecke K, Giaume C (2008). Astroglial metabolic networks sustain hippocampal synaptic transmission. Science.

[167] Panchin YV (2005). Evolution of gap junction proteins--the pannexin alternative. J Exp Biol.

[168] Baranova A, Ivanov D, Petrash N, Pestova A, Skoblov M, Kelmanson I, Shagin D, Nazarenko S, Geraymovych E, Litvin O (2004). The mammalian pannexin family is homologous to the invertebrate innexin gap junction proteins. Genomics.

[169] Bruzzone R, Hormuzdi SG, Barbe MT, Herb A, Monyer H (2003). Pannexins, a family of gap junction proteins expressed in brain. Proc Natl Acad Sci U S A.

[170] Thompson RJ, Zhou N, MacVicar BA (2006). Ischemia opens neuronal gap junction hemichannels. Science.

[171] Bargiotas P, Krenz A, Hormuzdi SG, Ridder DA, Herb A, Barakat W, Penuela S, von Engelhardt J, Monyer H, Schwaninger M (2011). Pannexins in ischemia-induced neurodegeneration. Proc Natl Acad Sci U S A.

[172] MacVicar BA, Thompson RJ (2010). Non-junction functions of pannexin-1 channels. Trends Neurosci.

[173] Dahl G, Keane RW (2012). Pannexin: from discovery to bedside in 11+/−4 years?. Brain Res.

[174] Le Vasseur M, Lelowski J, Bechberger JF, Sin WC, Naus CC (2014). Pannexin 2 protein expression is not restricted to the CNS. Front Cell Neurosci.

[175] Bruzzone R, Barbe MT, Jakob NJ, Monyer H (2005). Pharmacological properties of homomeric and heteromeric pannexin hemichannels expressed in Xenopus oocytes. J Neurochem.

[176] Qu Y, Misaghi S, Newton K, Gilmour LL, Louie S, Cupp JE, Dubyak GR, Hackos D, Dixit VM (2011). Pannexin-1 is required for ATP release during apoptosis but not for inflammasome activation. J Immunol.

[177] Bao L, Locovei S, Dahl G (2004). Pannexin membrane channels are mechanosensitive conduits for ATP. FEBS Lett.

[178] Sosinsky GE, Boassa D, Dermietzel R, Duffy HS, Laird DW, MacVicar B, Naus CC, Penuela S, Scemes E, Spray DC (2011). Pannexin channels are not gap junction hemichannels. Channels (Austin).

[179] Penuela S, Bhalla R, Gong XQ, Cowan KN, Celetti SJ, Cowan BJ, Bai D, Shao Q, Laird DW (2007). Pannexin 1 and pannexin 3 are glycoproteins that exhibit many distinct characteristics from the connexin family of gap junction proteins. J Cell Sci.

[180] Boassa D, Qiu F, Dahl G, Sosinsky G (2008). Trafficking dynamics of glycosylated pannexin 1 proteins. Cell Commun Adhes.

[181] Sandilos JK, Bayliss DA (2012). Physiological mechanisms for the modulation of pannexin 1 channel activity. J Physiol.

[182] Weilinger NL, Tang PL, Thompson RJ (2012). Anoxia-induced NMDA receptor activation opens pannexin channels via Src family kinases. J Neurosci.

[183] Bhalla-Gehi R, Penuela S, Churko JM, Shao Q, Laird DW (2010). Pannexin1 and pannexin3 delivery, cell surface dynamics, and cytoskeletal interactions. J Biol Chem.

[184] Billaud M, Sandilos JK, Isakson BE (2012). Pannexin 1 in the regulation of vascular tone. Trends Cardiovasc Med.

[185] Thompson RJ, Macvicar BA (2008). Connexin and pannexin hemichannels of neurons and astrocytes. Channels (Austin).

[186] Zoidl G, Petrasch-Parwez E, Ray A, Meier C, Bunse S, Habbes HW, Dahl G, Dermietzel R (2007). Localization of the pannexin1 protein at postsynaptic sites in the cerebral cortex and hippocampus. Neuroscience.

[187] Sandilos JK, Chiu YH, Chekeni FB, Armstrong AJ, Walk SF, Ravichandran KS, Bayliss DA (2012). Pannexin 1, an ATP release channel, is activated by caspase cleavage of its pore-associated C-terminal autoinhibitory region. J Biol Chem.

[188] Silverman WR, de Rivero Vaccari JP, Locovei S, Qiu F, Carlsson SK, Scemes E, Keane RW, Dahl G (2009). The pannexin 1 channel activates the inflammasome in neurons and astrocytes. J Biol Chem.

[189] Chekeni FB, Elliott MR, Sandilos JK, Walk SF, Kinchen JM, Lazarowski ER, Armstrong AJ, Penuela S, Laird DW, Salvesen GS (2010). Pannexin 1 channels mediate ‘find-me’ signal release and membrane permeability during apoptosis. Nature.

[190] Gulbransen BD, Bashashati M, Hirota SA, Gui X, Roberts JA, MacDonald JA, Muruve DA, McKay DM, Beck PL, Mawe GM (2012). Activation of neuronal P2X7 receptor-pannexin-1 mediates death of enteric neurons during colitis. Nat Med.

[191] Orellana JA, Froger N, Ezan P, Jiang JX, Bennett MV, Naus CC, Giaume C, Saez JC (2011). ATP and glutamate released via astroglial connexin 43 hemichannels mediate neuronal death through activation of pannexin 1 hemichannels. J Neurochem.

[192] Shestopalov VI, Slepak VZ (2014). Molecular pathways of pannexin1-mediated neurotoxicity. Front Physiol.

[193] Dvoriantchikova G, Ivanov D, Barakat D, Grinberg A, Wen R, Slepak VZ, Shestopalov VI (2012). Genetic ablation of Pannexin1 protects retinal neurons from ischemic injury. PLoS One.

[194] Murphy N, Cowley TR, Richardson JC, Virley D, Upton N, Walter D, Lynch MA (2012). The neuroprotective effect of a specific P2X(7) receptor antagonist derives from its ability to inhibit assembly of the NLRP3 inflammasome in glial cells. Brain Pathol.

[195] Elliott MR, Chekeni FB, Trampont PC, Lazarowski ER, Kadl A, Walk SF, Park D, Woodson RI, Ostankovich M, Sharma P (2009). Nucleotides released by apoptotic cells act as a find-me signal to promote phagocytic clearance. Nature.

[196] Adamson SE, Leitinger N (2014). The role of pannexin1 in the induction and resolution of inflammation. FEBS Lett.

[197] Liu F, Li Z, Li J, Siegel C, Yuan R, McCullough LD (2009). Sex differences in caspase activation after stroke. Stroke.

[198] Ikonomidou C, Turski L (2002). Why did NMDA receptor antagonists fail clinical trials for stroke and traumatic brain injury?. Lancet Neurol.

